# Production of orbital angular momentum states of optical vortex beams using a vortex half-wave retarder with double-pass configuration

**DOI:** 10.1038/s41598-022-10131-0

**Published:** 2022-04-11

**Authors:** Sarayut Deachapunya, Sorakrai Srisuphaphon, Sitti Buathong

**Affiliations:** 1 Department of Physics, Faculty of Science, Burapha University, ChonBuri, 20131 Thailand; 2 Thailand Center of Excellence in Physics, Ministry of Higher Education, Science, Research and Innovation, 328 Si Ayutthaya Road, Bangkok, 10400 Thailand; 3 Quantum and Nano Optics Research Unit, Burapha University, ChonBuri, 20131 Thailand

**Keywords:** Optics and photonics, Physics

## Abstract

Higher orders of orbital angular momentum states (OAMs) of light have been produced with a double-pass configuration through a zero-order vortex half-wave retarder (VHWR). This double-pass technique can reduce the number of VHWR plates used, thus reducing costs. The OAM states of the vortex beams are identified by the near-field Talbot effect. Polarization dependence of the vortex states can also be demonstrated with this VHWR using Talbot effect. Without using the Talbot patterns, this effect of the polarization on the vortex beam can not be recognized. A theoretical validation has also been provided to complement the experimental results. Our study gives an improved understanding of this approach to use a VHWR plate.

## Introduction

A vortex beam is a light beam that is characterized via a phase factor exp(i$\ell \phi$) around its phase singularity at the beam center, where $\phi$ is the azimuthal coordinate and $\ell$ is an integer representing topological charge. Since Allen et al.^1^ demonstrated in a typical laboratory in 1992 that optical vortex beams with helical phase-front possess orbital angular momentum (OAM) of $\ell \hbar$ per photon, considerable progress in fundamentals and applications of light vortices has been made. In particular, some of the fundamental research has been dedicated to exploring the polarization descriptions relevant to an optical vortex, such as polarization basis^2^. Characteristics of circularly polarized vortex beams were examined by adopting the decomposition of cylindrical bases^3^ and a special polarization grating^4^. Degenerate Stokes states were differentiated by a method associated with transformations of diffraction and polarization^5^. Additionally, studies on OAM light beams have shown promising applications in various fields. For instance, in the field of optical communication, infinite orthogonal OAM states of an optical vortex can be applied for mode division multiplexing, and this can be incorporated along with other existing multiplexing approaches such as polarization multiplexing^6^, and data encoding to implement a realization of very large capacity information transmission^7,8^. Twisted photons carrying OAM have also been utilized in high dimensional quantum communication^9^. In laser processing and lithography, due to its helical wavefront nature, a vortex light beam was employed to fabricate chiral nanostructures^7,10^ and micro-pipe structures^11^. In OAM imaging, super-resolution microscopy beyond the light diffraction limit, so-called stimulated emission depletion (STED), can be obtain by exploiting properties of zero intensity along the optical axis of a vortex beam having a low angular quantum number^12^. Investigation of interactions of OAM light with matter have also led to the optical manipulation of microparticles^13,14^. Optical vortices were coupled with optical tweezers to trap and force particles to rotate around the vortex singularity^15^. With the aid of a programmable spatial light modulator (SLM), holographic optical tweezers of OAM light were produced to capture and transport many particles simultaneously^16–18^. The light topography, so-called optical grinder, was recently generated by OAM carrying Laguerre-Gaussian beams and a SLM for optical trapping and size-selective particle sorting^19^.

For the creation of an optical vortex, numerous alternative methods have been reported^7,14,20^. They can be typically grouped into intra-cavity mode selection and extra-cavity conversion techniques. The intra-cavity method involves production of OAM fields via insertion of some optical components including a spiral phase plate^21,22^, a thin opaque disk (a stop)^23^, thin aluminum stripes^24^, or a tilted etalon^25^ into the laser resonator to increase the losses of undesired modes. On the other hand, the extra-cavity conversion mean is related to generation of an optical vortex outside the laser cavity using optical elements such as a SLM^26,27^, a forked grating^28^, cylindrical lenses^29^, and transmissive programmable metasurfaces^30^. Recently, polarization optics configuration known as a vortex half-wave retarder (VHWR) has been utilized to convert Gaussian laser beams into Laguerre-Gaussian modes with OAM^20,31^. Xiujian Li et al. illustrated that high-order cylindrical vector beams were realized by cascading multiple VHWRs^32^. Furthermore, VHWR was used to generate a vortex beam from a partly incoherent light such as a light-emitting diode (LED)^33^, to help trap metallic particles^34^ and to measure full polarization states of light^35^ and optical rotation effect^36^.

There are a variety of approaches to detect OAM light. The study of creation and detection of optical modes with spatial light modulators was reported^37^. An excellent method for measuring the vortex and orbital angular momentum spectra with a single cylindrical lens was also reported^38^. The interference and diffraction characteristics of optical vortices have been utilized for measurements of the OAMs of light. The interferometric techniques involving a shearing interferometer and an inverted field interferometer were shown to determine the properties of ultrafast vortices^39,40^. An improved multipoint interferometer was manifested to probe the vortex beams with high OAM charge^41^. The self-referenced interference method using a Mach-Zehnder interferometer was used for detection of the magnitude and sign of the vortex numbers^42^. The Fraunhofer diffraction patterns of vortex beams through special apertures such as a square aperture^43^, a diamond-shaped aperture^44^, an isosceles triangular aperture^45^, a binaural circular aperture^46^ were also studied for OAM determination. Another approach is to make use of the near-field diffraction phenomenon known as the Talbot effect^47^ to characterize optical vortices and their topological charges^26,27^. Two-dimensional Talbot effect of the optical vortices has also been reported^48^. It was demonstrated later that the Talbot patterns resulting from an overlapping grating configuration can provide high optical vortex detection efficiency^31,49^. Not only has the Talbot effect been shown to help determine the OAM of light, but it also has other potential applications^50–53^.

In this paper, a new economical double-pass configuration through VHWRs is proposed for the creation of optical vortices of several OAM orders. These OAM beams are detected and distinguished using the high-contrast Talbot patterns formed via the overlapping grating setup^31,49^. The influence of the vortex polarization states of light on the Talbot effect was also investigated. The simulated results are in good agreement with the experimental observations, validating our theoretical description.

## Theory and methodology

**Figure 1:**
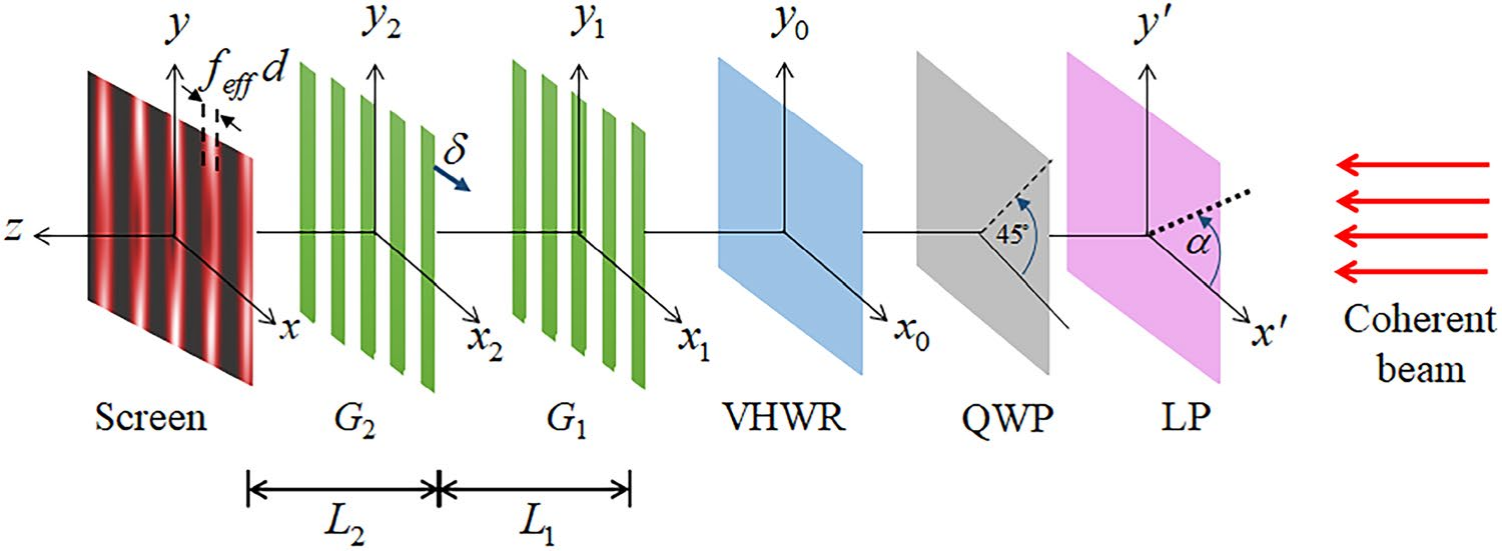
A coherent beam falls on a linear polarizer (LP) and propagates through a quarter-wave plate (QWP) to a vortex half-wave retarder (VHWR) for generating an optical vortex (OV). The angle $\alpha$ indicates the fast axis of the LP, used to change the input polarization of the laser beam, while the fast axis of QWP is fixed at 45$^\circ$. The OV diffracts through the two overlapping gratings ($G_1$ and $G_2$) with period *d*. A transverse shift by $\delta$ yields an interference pattern with small bright fringe width due to the effective open fraction $f_{eff}$.

In this section, we present a theoretical approach for studying the vortex beam and the effects of the polarization states on the vortex beam utilizing the Talbot effect. According to the setup diagram in Fig. 1, the incoming coherent beam encounters a linear polarizer (LP) with a polarization axis indicated by angle $\alpha$ on the $x'y'$-plane. Later, a quarter-wave plate (QWP) transforms that beam to be circularly polarized (CP), which will act as the input polarization. Subsequently, the obtained CP beam is transformed by the VHWR to an optical vortex (OV) with orbital number -$\ell$ or +$\ell$ depending on the input polarization. Behind the VHWR, the Jones vector of the transmitted beam can be expressed as^20^

1 $$\begin{aligned} \tilde{E}= & \,R(x_0,y_0)\left( \begin{array}{ll} \cos 2\theta &{} \sin 2\theta \\ \sin 2\theta &{} -\cos 2\theta \\ \end{array} \right) \cdot \frac{1}{\sqrt{2}} \left( \begin{array}{ll} 1 &{} -i\\ -i &{} 1 \\ \end{array} \right) \cdot \left( \begin{array}{l} \cos \alpha \\ \sin \alpha \\ \end{array} \right) \nonumber \\\equiv &{} \,R(x_0,y_0)~\tilde{V}_{\ell }\cdot \tilde{Q}\cdot \tilde{P}. \end{aligned}$$

The function $R(x_0,y_0)$ for characterizing the spatial distribution involves the OV radius on the $x_0 y_0$-plane. Here, $\tilde{V}_{\ell }$ symbolizes the VHWR Jones matrix with fast axis direction at an azimuth angle $\theta$. The middle matrix $\tilde{Q}$ is according to the QWP Jones matrix with fast axis in $45^\circ$ orientation referred to the horizontal direction ($x'$), while the Jones vector $\tilde{P}$ stands for the linearly polarized beam.

The angle $\theta$ is related to the VHWR azimuth angle $\phi _0$ by $2\theta =\ell \phi _0+2\sigma$, where $\sigma$ denotes the fast axis direction when $\phi _0=0$^20^. Therefore, using $\phi _0=\arctan (y_0/x_0)$ and taking $\sigma =0$, we can input OAMs wave function into $\tilde{E}$ as follows:

2 $$\begin{aligned} \tilde{E}= & \,\frac{1}{\sqrt{2}}\left( \begin{array}{ll} \varphi _{-\ell }(x_0,y_0) &{} -i \varphi _{+\ell }(x_0,y_0)\\ i \varphi _{-\ell }(x_0,y_0)& -\varphi _{+\ell }(x_0,y_0) \\ \end{array} \right) \cdot \left( \begin{array}{l} \cos \alpha \\ \sin \alpha \\ \end{array} \right) , \end{aligned}$$

where $\varphi _{\pm \ell }(x_0,y_0) = R(x_0,y_0) e^{\pm i \ell \phi _0}$ is the wave function for OAMs with the topological charge number $\pm \ell$^54^.

Nevertheless, to identify the OAMs with diffraction patterns, the propagation of $\varphi _{\pm \ell }(x_0,y_0)$ has to be involved^5^. According to our previous report^31^, we have applied adjustable combination gratings ($G_1$ and $G_2$ in Fig. 1) for generating the near-field Talbot effect to determine both order and charge of the OAMs. We employed the Gaussian function $R(x_0,y_0)=\exp \{(-1/w^2)(x_0^2+y_0^2)\}$, with which the Fresnel-integrals can be evaluated analytically^26^ over the distances $L_1$ and $L_2$ through the two gratings $G_1$ and $G_2$. Here, *w* is the Gaussian radius of the vortex dimension for adjusting theoretical simulations to the experimental results. The wave function that corresponds to near-field diffraction with the two overlapping gratings is given by

3 $$\begin{aligned} \psi _{\pm \ell }(x,y)= & {} \sum _{n_1,n_2}A_{n_1}A_{n_2}F^{|\ell |}_{n_1,n_2}(x,y) \nonumber \\&\times \exp \{\beta (w)|F_{n_1,n_2}(x,y)|^2-2\pi ig_{n_1,n_2}(x)\}, \end{aligned}$$

where

4 $$\begin{aligned} F_{n_1,n_2}(x,y)= & \,(x+(2n_1+n_2)d) \pm i y,\nonumber \\ \beta (w)= & \,\frac{i \pi }{d^2} \Big (\frac{d^2+i \pi w^2}{2d^2+i \pi w^2}\Big ), \nonumber \\ g_{n_1,n_2}(x)= & \,\frac{n_1 x}{d}+ n_2 f_{eff}+ n_1(n_1+n_2). \end{aligned}$$

The Fourier components $A_{n_j} =\sin (n_j \pi f) / n_j \pi$ are for periodic regular grating with open fraction $f=0.5$^55^. These components associate $A_{n_j}$ with the grating transmission functions $G_j(x_{j})=\sum _{n_j}A_{n_j} \exp \{2 \pi i n_{j} x_{j}/d\}$ where $j=1, 2$ with *d* is the grating period. A relative transverse shift by $\delta$ between the two gratings (as shown in Fig. 1) gives a reduced effective open fraction $f_{eff}<0.5$. In the near-field regime on assigning both distances $L_1$ and $L_2$ identical to $L_T=d^2/\lambda$, the self-image of $G_1$ diffracted through grating $G_2$ yields the second self-image with bright fringe width $\delta =f_{eff}d$^49^. All of the independent factors and phase constants for the diffraction patterns have been ignored.

Lastly, according to the diffraction from the plane $x_1y_1 \rightarrow x_2y_2\rightarrow xy$, we replace $\varphi _{\pm \ell }(x_0,y_0)$ in Eq. (2) with $\psi _{\pm \ell }(x,y)$ and this yields the intensity distribution $I_{|\ell |}(x,y,\alpha )$ corresponding to the interference pattern as

5 $$\begin{aligned} I_{|\ell |}(x,y,\alpha )=\tilde{E^\dag }\tilde{E}=|\psi _{-\ell }(x,y)|^2\cos ^2\alpha + |\psi _{+\ell }(x,y)|^2\sin ^2\alpha . \end{aligned}$$

The individual interference pattern with OAMs having $-\ell$ appears when $\alpha$= $0^o$, which corresponds to the input polarization of right-handed circularly polarized (RHCP) light. On the other hand, if $\alpha$= $90^o$ or the input light is left-handed circularly polarized (LHCP), then the $+\ell$ state is acquired. The case $0^o<\alpha <90^o$ results in a mixed interference pattern of both above states.

## Experimental setup

**Figure 2:**
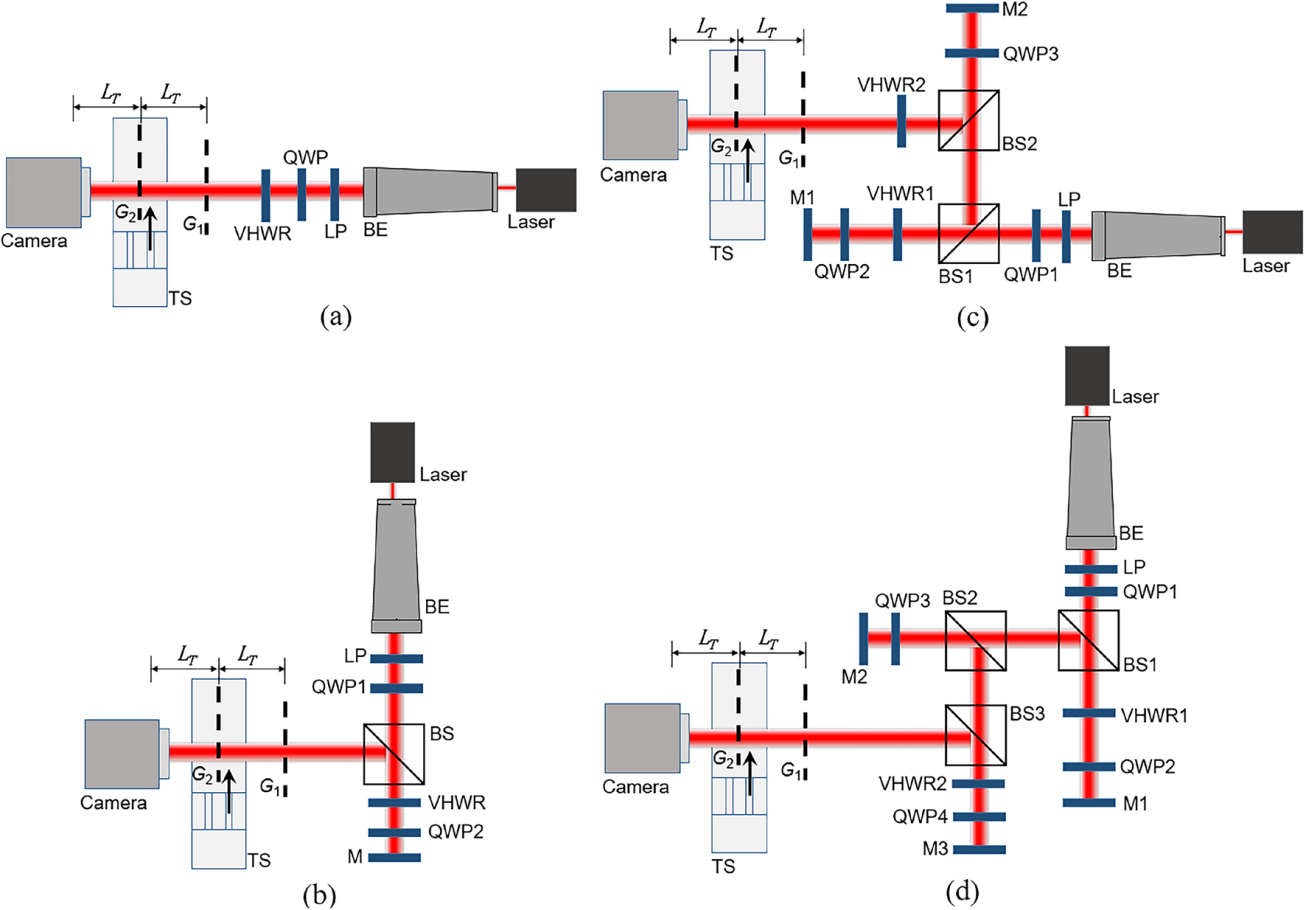
Experimental demonstration of production and identification of the vortex beam **(a)** $\ell =\pm 1$, **(b)** $\ell =\pm 2$, **(c)** $\ell =\pm 3$, and **(d)** $\ell =\pm 4$. The double-pass configuration applies for higher orders of the vortex beam. The Talbot probe, which consists of a double-grating system ($G_1$ and $G_2$) and a CCD camera, was used to measure and identify the order and sign of the vortex beam. Please see the text for details.

We tested our idea by producing vortex beams with $\ell =\pm 1, \pm 2, \pm 3, \pm 4$ using the setups shown in Fig. 2. For $\ell =\pm 1$, we simply used a single-pass configuration^20,31,33^ (Fig. 2a). A 780 nm stabilized laser (Laser, Ondax, laser diode CP-780.25-PLR-140, CP) was used as the coherent light source. A beam expander (BE, GBE15-A, Thorlabs) allows expanding the laser beam to about 15 mm diameter to cover all grating lines. Circularly polarized light with both left- or right-handed circular polarization can be produced by a polarizer (LP, LPVIS100-MP2, Thorlabs) and a quarter-wave plate (QWP, WPQ10M-780, Thorlabs). These polarization states are required for producing the OV beam using a VHWR (VHWR, WPV10L-780, Thorlabs). Namely, the left-handed circular polarization (LHCP) produces the vortex with +$\ell$, while the right-handed circularly polarized (RHCP) light will produce the opposite ($-\ell$). The Talbot effect with a diffraction grating ($G_1$)^47,55^, together with the use of the grating mask ($G_2$)^49^, made it possible to clearly measure the order and sign of the vortex beam at the same time^26,27^. Both are normal binary gratings (200 $\upmu$m period, chromium on glass, Edmund Optics Inc.). A translation stage (TS, MTS50/M-Z8, Thorlabs) can be adjusted for an arbitrary effective open fraction $f_{eff}$^31,49^ of the Talbot patterns by moving the second grating ($G_2$) transversely in order to obtain sharp Talbot images for detection by a USB 2.0 CMOS camera (Camera, DCC1545M, Thorlabs). The distance between the first ($G_1$) and second ($G_2$) grating was set to one Talbot distance ($L_T$), as was the distance between the second grating and the camera. These distances can also be set at multiples of the Talbot length.

**Figure 3:**
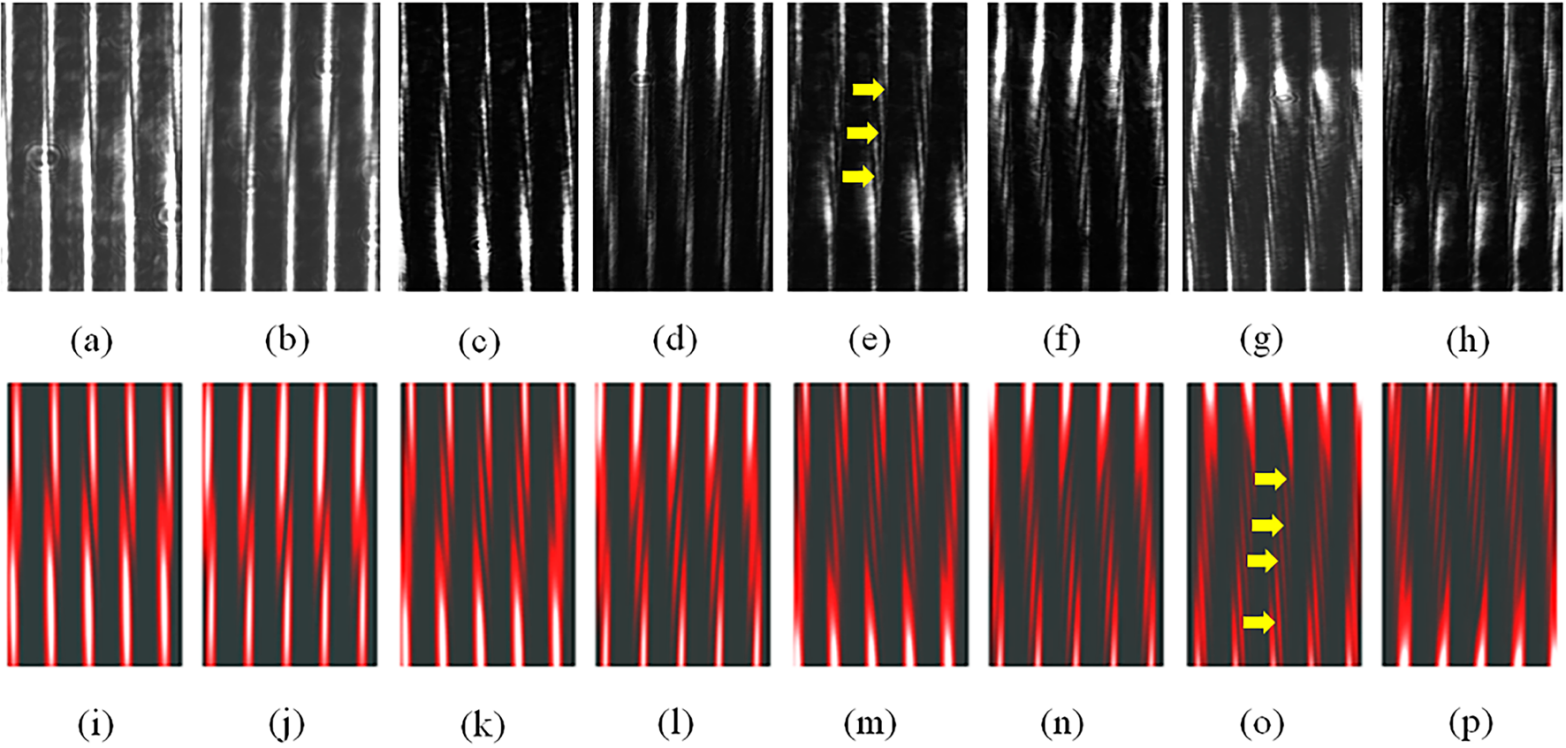
Talbot patterns experimentally recorded with: **(a)** $\ell =+1$, **(b)** $\ell =-1$, **(c)** $\ell =+2$, **(d)** $\ell =-2$, **(e)** $\ell =+3$, **(f)** $\ell =-3$, **(g)** $\ell =+4$, and **(h)** $\ell =-4$. Theoretical simulations of $I_{|\ell |}(x,y,\alpha )$ presented in **(i)**–**(p)** correspond to the conditions of **(a)**–**(h)** according to Eq. (5) with $f_{eff}=0.25$, and $w=5d$. The arrows in **(e)** and **(o)** point to examples of the tilted dark stripes in the middle of the interference fringes, used to identify the vortex beam. Please see the text for details.

For higher orders of the vortex beam $\ell =\pm 2, \pm 3, \pm 4$, the double-pass configuration was applied as seen in Fig. 2b–d, respectively. In Fig. 2b, the vortex beam with $\ell =\pm 2$ can be produced using only one VHWR plate. The mirror (M, BB1-E03, Thorlabs) was used to reflect light back onto the backside of the VHWR to create this $\ell =\pm 2$. As this VHWR plate also changes the polarization of the light to the opposite of that of the input, the second quarter-wave plate (QWP2) must be used to return the polarization state to the initial state as the one passing through the first quarter-wave plate (QWP1). This QWP2 can be set to any angle. For $\ell =\pm 3$ shown in Fig. 2c, the first VHWR (VHWR1) and second VHWR (VHWR2) were aligned as the double-pass and single-pass configuration, respectively. This combination creates the vortex beam with $\ell =\pm 3$. Again, three quarter-wave plates (QWP1, QWP2, QWP3) were used to compensate for the polarization of the light. The case $\ell =\pm 4$ requires two double-pass VHWR plates (VHWR1, VHWR2) in the setup of Fig. 2d. At the Talbot image detection, the second grating ($G_2$) was adjusted in the transverse direction to make the image as sharp as possible.

**Figure 4:**
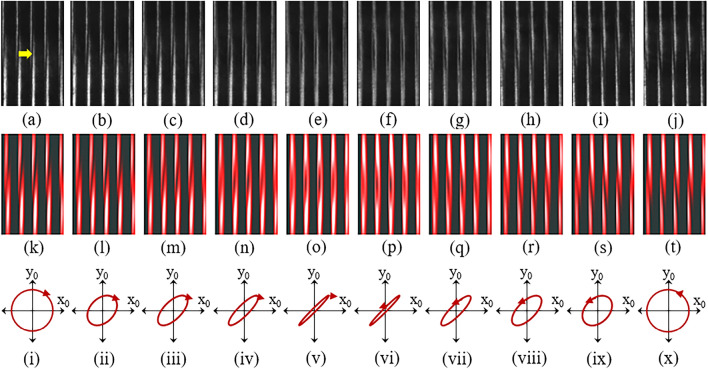
Experimental Talbot patterns with the vortex beam recorded with: **(a)** $\alpha$= $0^\circ$, **(b)** $\alpha$= $10^\circ$, **(c)** $\alpha$= $20^\circ$, **(d)** $\alpha$= $30^\circ$, **(e)** $\alpha$= $40^\circ$, **(f)** $\alpha$= $50^\circ$, **(g)** $\alpha$= $60^\circ$, **(h)** $\alpha$= $70^\circ$, **(i)** $\alpha$= $80^\circ$, and **(j)** $\alpha$= $90^\circ$. Theoretical simulations of $I_{|\ell |}(x,y,\alpha )$ presented in **(k)**–**(t)** correspond to the conditions of **(a)**–**(j)**, respectively, according to Eq. (5) with $f_{eff}=0.25$, and $w=5d$. The results show the rotation of the tilted dark stripe, indicated by the arrow, due to the input polarization. Cases (i)–(x) show the input polarization behind QWP in Fig. 2a from right-handed circular polarization (i) to left-handed circular polarization (x), referred to the cases used in **(a)**–**(j)**, respectively.

Polarization dependence of the vortex beam was also studied using the setup in Fig. 2a. The LP was rotated from horizontal ($\alpha$= $0^\circ$) to vertical polarization ($\alpha$= $90^\circ$) in steps of 10$^\circ$, while the QWP was fixed at an angle of 45$^\circ$. This causes the input polarization to change from right-handed circular (Fig. 4i) to elliptical (Fig. 4ii–ix) and to the final state of left-handed circular polarization (Fig. 4x). The Talbot probe was again used to measure the vortex beam for each angle of the LP.

## Results and discussion

Figure 3 shows the results of the OV beam of $\ell =\pm 1, \pm 2, \pm 3, \pm 4$ produced by the single-pass and double-pass configurations. The orders ($\ell$) and signs (±) of the OAMs can be explored from the trails of the tilted dark stripes in the middle of the interference fringes, indicated by the arrows in Fig. 3. The orders, $\ell$ can be determined by the number of the tilted dark stripes and the signs, ± obtained from the tilt direction of the dark stripes. The theoretical simulations (Fig. 3i–p) are very consistent with the experimental results (Fig. 3a–h). Therefore, our theoretical approach can match experiments with higher OAMs.

We have demonstrated in detail the use of a VHWR plate. The spin-orbit beam has been studied with varied polarization states of this OV beam. The input polarization required for use of the VHWR was varied from right-handed circular to elliptical polarization, and finally to left-handed circular polarization, by rotating the optical axis of LP in Fig. 2a by adjusting $\alpha$ from horizontal ($\alpha$= $0^\circ$) to vertical polarization ($\alpha$= $90^\circ$) in steps of 10$^\circ$. Again, the tilted dark stripe in the middle of the interference fringes, indicated by the arrow in Fig. 4, was used to indicate the effects. The results show prominently the rotation of the tilted dark stripe affected by the input polarization. These results demonstrate the use of a VHWR plate and theoretical foundations for its applications.

## Conclusion

Here, we have shown that the double-pass configuration through a VHWR can generate an OV beam across higher orders ($\ell >\pm 1$) of OAMs. The Talbot effect used as probing method is excellent for measuring OAMs of the OV beam. The modulation of the OV beam according to the input polarization of the VHWR has also been realized using this Talbot probe. Without using the near-field Talbot effect, the influence of the polarization of the OV beam can not be recognized. Our theory was demonstrated to explaining and predict experimental results extremely well. This study provides improved understanding of the use of a VHWR plate, offering possibilities for further applications.

## Data Availability

The data that support this study results are available from the corresponding author upon reasonable request.
